# Three Successful Cases of Tracheotomy

**Published:** 1878-07

**Authors:** D. A. Walden

**Affiliations:** Beatrice, Neb.


*Three Cases of Tracheotomy, and Recovery of Each.*

Case I. Foreign substance in trachea.

On the morning of October 2d, 1877, I was called in haste to see Willie D., a robust, healthy boy, aged two years and six months. The patient was quiet, but his respirations were very stridulous, especially on expiration. A physician had been called and pronounced the case croup ; but the usual remedies employed failed to hinder the progress of the disease. From the history of the case the difficulty encountered on expiration convinced me at once that there was some foreign substance in the trachea. As the child seemed in imminent danger of death from suffocation, I at once proposed tracheotomy to the parents, as the only means of relief.

After summoning another physician, who readily agreed with me as to the diagnosis, we proceeded at once to operate, chloroform being the anæsthetic used. An incision was made and a kernel of corn removed from the trachea. The wound was then closed by sutures, and covered with carbolized dressings. A rapid recovery followed. The operation was attended with but very little hæmorrhage.

Case II. Foreign substance in trachea.

September 10th, 1875, I was called to see Emma W., aged five years, with symptoms similar to those described above. I at once proceeded to open the trachea below the isthmus of the thyroid gland, and extracted a large bean. I dressed the wound neatly and the patient made a recovery without any bad symptoms.

Case III. Croup.

I was called in November, 1877, to see Willie H., aged five years, and found the patient suffering from pseudo-membranous croup. He had been sick about four days, and was under the care of another physician. As death was inevitable unless something was done to procure relief, we advised immediate tracheotomy as the only means of saving life. The parents of the child objected to the performance of the operation, as they thought it would be productive of instant death ; but after better thought, and becoming persuaded that there was no other possible hope for the child, they consented to have the operation performed on the next morning. With the aid of an assistant, I proceeded at once to operate. The incision had no sooner been made than the child threw out a large amount of false membrane. I then cauterized the wound and inserted the tube. Chloroform and ether were employed as anæsthetics.

I was called again in a couple of hours, and found the breathing very difficult. On removing the tube, a large piece of membrane was thrown out of the opening. I then re-inserted the tube and did not remove it for three days. Convalescence then set in, and the patient made a complete recovery in the course of weeks.

As to the time when tracheotomy is to be performed, we urge an early operation, not deferring it until the patient is asphyxiated. In my opinion, the operation of tracheotomy would be the means of saving many lives, if physicians and surgeons would resort to it sooner, as I regard the operation when properly performed, attended with comparatively little danger to the patient.

The following means of diagnosis, which we take from "Watson's Practice," if observed will prove a great help to the average practitioner: "The symptoms that imperatively cry out for the operation are, increasing pallor, cold sweats, lividity of skin and lips, extreme feebleness of pulse, and sinking in, during every effort to inspire, of the costal spaces, of the hollows above the collar bones and the breast bone, and of the flexible lower margins of the thorax."

D. A. Walden, M. D.

Beatrice, Neb.

